# Pelvic Organ Prolapse in Ehlers-Danlos Syndrome

**DOI:** 10.1155/2023/6863711

**Published:** 2023-02-23

**Authors:** Azadeh Nazemi, Katherine Shapiro, Shavy Nagpal, Nirit Rosenblum, Benjamin M. Brucker

**Affiliations:** Department of Urology, New York University Grossman School of Medicine New York, NY, USA

## Abstract

Ehlers-Danlos syndrome (EDS) is a hereditary tissue and collagen synthesis disorder that can predispose patients to gynecologic and obstetric complications. Female patients often suffer from bothersome pelvic floor disorders, but due to the medical complexity of EDS, special considerations are needed for the treatment of pelvic organ prolapse and associated incontinence. In this paper, we present three unique cases of pelvic organ prolapse (POP) in EDS patients and delve deeper into the multidisciplinary approach involving urogynecology, rheumatology, physiatry, gastroenterology, and anesthesiology required to appropriately manage this condition.

## 1. Introduction

Ehlers-Danlos syndrome (EDS) has a prevalence of about 1 : 5000 and falls under a group of hereditary disorders targeting connective tissue and collagen synthesis [1]. Twelve different subtypes affect collagen production and processing pathways. The hypermobile EDS variant (type III) is the most common subtype and thought to be inherited in an autosomal dominant fashion with no known genetic mutation. While commonly associated with hyperextensible skin, hypermobile joints, and hematological manifestations including increased tendency to bleed, defects in collagen biosynthesis at a molecular level predispose to gynecologic and obstetric complications [2].

Female patients with EDS often have bothersome pelvic floor disorders including pelvic organ prolapse (POP) and stress urinary incontinence (SUI) [2–4]. These disorders can present at a young age without the typical risk factors such as pregnancy and delivery or menopause. The management of POP in patients with EDS involves a dedicated and multidisciplinary approach which is rarely reported in the literature.

## 2. Case Presentations

### 2.1. Case 1

A 19-year-old G0P0 female with Ehlers-Danlos syndrome (EDS) type III and postural orthostatic tachycardia syndrome (POTS) presented with a history of chronic pelvic pain, symptomatic vaginal bulge/pressure, and pelvic muscle spasms. She had seen multiple physicians at different institutions for similar symptoms over one year.

The patient was initially managed conservatively with pain control for pelvic floor muscle spasms with vaginal diazepam suppositories. She trialed a vaginal pessary but stopped due to vaginal discharge, bleeding, and discomfort. She participated in pelvic floor physical therapy and noted improvement in muscle tightness; however, she complained of a persistent vaginal bulge secondary to prolapse.

**Figure 1:**
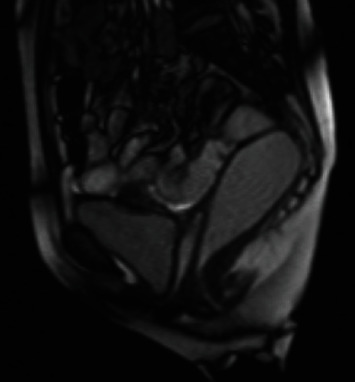
MRI defecography demonstrating anterior compartment prolapse. Anterior compartment descends 1.2 cm below the pubococcygeal line (PCL) with defecation consistent with mild anterior prolapse.

She was referred to a female pelvic medicine and reconstructive surgery (FPMRS) surgeon. Pelvic Organ Prolapse Quantification system (POP-Q) on evaluation by an FPMRS surgeon revealed Aa -3, Ba -3, C -5, D -7, Ap -3, Bp -3, Pb 3, Gh 2, and TVL 9. A dynamic pelvic floor MRI revealed mild anterior vaginal wall prolapse with urethral hypermobility but did not reveal significant uterine prolapse in the supine position (Figure 1). The patient remained very bothered by vaginal pressure and discomfort attributed to uterine descent and was interested in proceeding with surgery. The options of robotic uterosacral ligament suspension and sacrohysteropexy were discussed including the pros/cons of each, and the patient expressed that she remained uninterested in future pregnancy, deciding to pursue robotic sacrohysteropexy. Anesthesia was made aware of the case beforehand for preoperative optimization of her POTS, including adequate hydration, and intraoperative avoidance of hyperextension of her neck. Ultimately, sacrohysteropexy was accomplished using a 4 cm by 15 cm strip of Polyform mesh that was secured to the posterior cervix using four 2-0 Gore-Tex sutures, tensioned to the sacral promontory, then sequentially secured to the anterior longitudinal ligament using three 2-0 Gore-Tex sutures.

**Figure 2:**
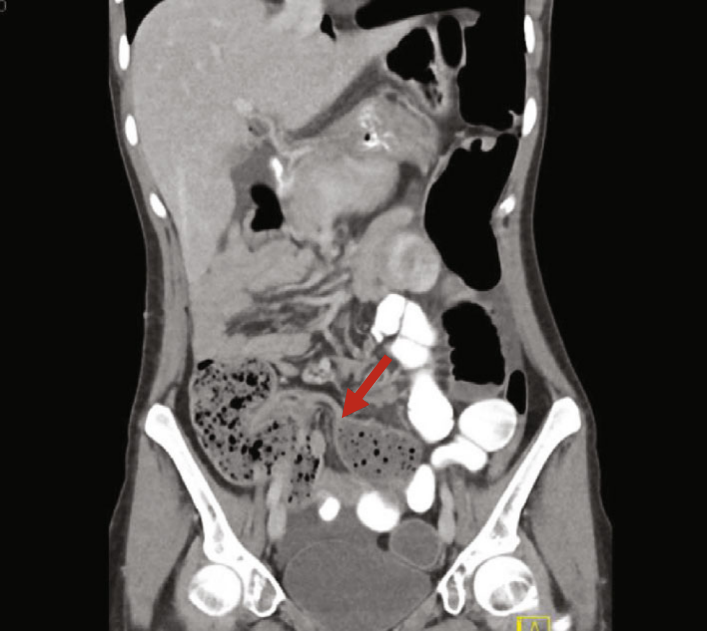
CT of the abdomen and pelvis showing with the red arrow indicating a transition point just proximal to the terminal ileum.

Four days postoperatively, the patient had persistent nausea, vomiting, and abdominal pain concerning ileus. A CT scan of the abdomen and pelvis revealed a probable small bowel obstruction with a transition point for which she was taken back to the operating room after consultation with general surgery (Figure 2). A robotic exploration revealed no evidence of significant transition point or bowel obstruction, confirming diffuse ileus with the absence of obstruction. The patient recovered well and followed up at three and six months with improvement in vaginal bulge and pelvic pain.

### 2.2. Case 2

A 38-year-old G2P2 female with Ehlers-Danlos syndrome type III, anxiety, asthma, and Breast Cancer Gene 1- (BRCA1-) positive status with prophylactic bilateral mastectomy, was referred to an FPMRS surgeon for an eight-year history of vaginal prolapse and a sharp burning pain in the pelvis. She had two atraumatic vaginal deliveries and her POP-Q revealed Aa 0, Ba 0, C -4, D -6, Ap 0, Pb 2, Gh 4, and TVL 7. She also noted urinary leakage with sneeze, incomplete bladder emptying, and the need to stimulate the clitoris to initiate urination. In addition, she complained of constipation with incomplete fecal emptying.

Due to her BRCA gene, she declined conservative management of her prolapse and chose to proceed with risk-reducing robotic total hysterectomy and bilateral salpingo-oophorectomy with concurrent sacrocolpopexy and retropubic midurethral sling to address her stress urinary incontinence. Sacrocolpopexy was recommended as the optimal approach given the extent of her prolapse symptoms. For the sacrocolpopexy, a type 1 lightweight polyproline Y shape graft was cut to 5 cm anteriorly and 7 cm posteriorly and secured in place with five 2-0 Gore-Tex sutures on each side.

**Figure 3:**
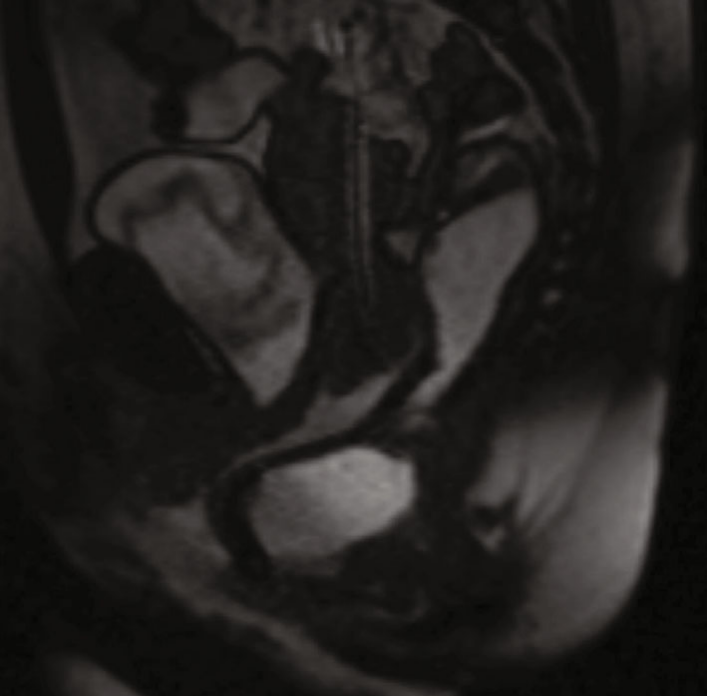
MR defecography demonstrates severe (>6 cm) anorectal junction descent during maximal strain. Severe (>4 cm) posterior vaginal wall prolapse is also noted. Of note, the patient did not defecate during the exam.

Preoperatively, the patient was referred to a colorectal surgeon to assess if rectopexy would aid in her bowel symptoms. An MR defecography showed severe posterior vaginal wall prolapse without rectal prolapse and complete pelvic floor descent with straining (Figure 3). As such, they did not recommend rectopexy, instead concurrent posterior vaginal wall prolapse repair was performed at the time of surgery. Postoperatively, based on recommendations from the colorectal surgery, she was referred for pelvic floor physical therapy for her pelvic floor descent to improve her fecal complaints. At her one-month follow-up appointment, the patient was very happy with her repair and had no complaints of leakage and improved bowel function.

### 2.3. Case 3

A 52-year-old G2P2 female with Ehlers-Danlos syndrome type III and symptomatic prolapse was referred to an FPMRS surgeon for urgency urinary incontinence. Her vaginal deliveries were atraumatic. She had been managed conservatively with a vaginal pessary for symptomatic prolapse but still had bothersome symptoms of a vaginal bulge. POP-Q revealed posterior vaginal wall prolapse and significant uterine descent with Aa -2, Ba -2, C -3, D -5, Ap 0, Bp 0, Pb 2, Gh 3, and TVL 8. Video urodynamics revealed normal bladder capacity and compliance with normal pressure voiding and no detrusor overactivity; stress urinary incontinence was present. She underwent robot-assisted supracervical hysterectomy, bilateral salpingo-oophorectomy, sacrocolpopexy, posterior vaginal wall prolapse repair, and retropubic midurethral sling placement. For the sacrocolpopexy, a type 1 lightweight polyproline Y shape graft was cut to 8 cm on both the anterior and posterior leaflets, then secured in place with six 2-0 Gore-Tex sutures on the anterior aspect and five on the posterior aspect. The long arm of the graft was secured to the anterior longitudinal ligament using three Ethibond sutures. At her 22-month follow-up, the patient remained satisfied and did not complain of bulge, with her posterior compartment well supported.

## 3. Discussion

Urinary incontinence and pelvic organ prolapse can be due to numerous etiologies including parity, age, ethnicity, estrogen deficiency, and smoking [1]. Connective tissue disorders have a higher prevalence in women (73-89%) compared to men and have also been shown to predispose individuals of these conditions [5]. The median age at diagnosis of EDS in women is commonly in the late twenties or early thirties. There is no test for hypermobile EDS, rather the diagnosis involves looking for joint hypermobility, signs of faulty connective tissue throughout the body (e.g., hernias or prolapses), a family history of the condition, and musculoskeletal problems.

From the limited literature, there are a wide range of reported pelvic floor symptoms in patients with connective tissue disorders, including urinary incontinence (38 to 60%), POP (13 to 75%), pelvic pain (13 to 75%), dyspareunia (30 to 77%), fecal incontinence (2 to 19%), and rectal prolapse (2 to 16%) [5]. To garner an updated understanding of pelvic floor symptoms, Kciuk et al. evaluated 1303 female participants through an online questionnaire to understand the obstetric and gynecologic experiences of cisgender women with EDS [5]. Sixty percent of participants reported SUI, 54% reported urgency urinary incontinence, 24% reported fecal incontinence, 21% reported POP, and 50% percent reported sexual dysfunction, with dyspareunia in 36%. Pelvic pain was also reported in 71% of participants. The majority of perioperative studies in patients with hereditary connective tissue disorders focus on orthopedic or vascular procedures [2]. One study assessed postoperative outcomes after surgery for POP or SUI and found that after adjusting for age, there is no significant difference in complication rates between patients with hereditary disorders of connective tissue and controls [2].

Unfortunately, studies of the effect on and management of POP and incontinence among women with these disorders are lacking [1]. Treatment for POP in patients with EDS requires a multidisciplinary approach, which is detailed below based on experience at a tertiary referral center.

Rheumatologists are often the first physicians involved with caring for EDS patients. Unfortunately, there is no single drug therapy to treat the pain and other comorbidities in patients with EDS [6]. Instead, therapy is often guided by the patient's symptomatology. Nonsteroidal anti-inflammatory drugs are the first line for pain, with ibuprofen being most effective and best tolerated [6]. While neuropathic modulators including tricyclic antidepressants, anticonvulsants, serotonin, and norepinephrine reuptake inhibitors are traditionally useful for centralized pain, their use in this patient population is limited secondary to 47% of patients noting adverse effects including worsening dysautonomia [6]. For patients with specific pelvic pain not responsive to oral analgesia or conservative management, injection with long-acting corticosteroids can be used [7].

Involving a physiatrist for patients with EDS who have POP is a key component of general management. Pelvic girdle pain is common and is a result of altered biomechanics throughout the pelvis [7]. Physiotherapy attempts to restore the balance of the pelvis by strengthening, stretching, improving flexibility, and stabilizing the soft tissues [7]. This helps improve pain within the soft tissues themselves and improves the biomechanics of the pelvis [7]. A randomized control trial has shown that a six-week generalized program improving strength and fitness resulted in significant improvements in pain scores and improvement in self-esteem [8, 9]. Another study showed specifically that patients with pelvic girdle pain respond well to targeted physical therapy [6]. There is no specific time frame after which therapy is considered effective, as training is often riddled with physical and mental setbacks, but the basic approach includes strength training, proprioceptive exercises, and gentle stretching [10].

In terms of initial treatment, patients often prefer to start with conservative management. This is not always successful, as one survey found that 75.5% of patients that trialed supportive appliances, such as pessaries, reported no improvement in symptoms. Meanwhile, 80.5% of patients who underwent surgery reported improvement in symptoms [5]. However, patients with hereditary connective tissue disorders require special consideration in surgical planning due to tissue characteristics and increased propensity to bleed. Anatomic recurrence of POP did not differ between patients with EDS and those without [2]. Additionally, the rate of postoperative complications did not differ between those with connective tissue disorders and controls [2].

No guidelines exist for general or regional anesthetic management of patients with EDS, which lends itself to careful consideration when developing the anesthetic plan [11]. As such, a thorough preoperative evaluation is essential to a successful and uneventful anesthesia experience [12]. From an anesthetic perspective, there are specific cardiovascular and airway considerations in the EDS population. Dysautonomia in the form of POTS, as seen in the first case presented here, is common, causing orthostatic hypotension secondary to abnormal aortic baroreceptors [12]. This results in symptomatic dizziness, nausea, palpitations, fatigue, and tachycardia. When combined with anesthesia, which causes peripheral vasodilation and hypotension, this can cause worsening dysautonomia furthering hemodynamic instability [12].

Additionally, hypermobility affects cartilaginous tissues, including the trachea, larynx, and skin. This poses difficulties with intubation secondary to the potential collapse of fibroelastic tissues in the trachea, cervical spine hypermobility or instability, or spontaneous pneumothorax [12]. Anesthesiologists should take care to avoid excessive neck extension and mouth opening during intubation [11]. For ventilation settings, using pressure control can avoid higher inspiratory positive pressures which might induce pneumothorax [11].

Specific surgical consideration due to tissue characteristics must be taken into account when planning to operate. Intraoperatively, the tissue may be friable and fragile making it difficult to hold suture material, resulting in diffuse- and difficult-to-control bleeding [13]. Gentle handling of tissues during surgery can help avoid injury caused by the reduced ability to resist mechanical forces [14]. Basic wound closure principles of preventing hematomas and performing tension-free closures are essential in avoiding cutaneous complications like wound dehiscence, poor suture holding, and wide scar formation [13]. Delaying or avoiding dressing changes minimizes frequent trauma to healing tissues and helps avoid overstretching of the skin [14]. Additionally, increased periods of immobilization and rest to healing tissue allows for a rest period during initial healing stages that may reduce chances of wound breakdown [14]. Appropriate preoperative counseling is imperative for this patient population. It is important to inform them that the overall success and failure rates of these procedures are not quoted in Ehlers-Danlos patients and that due to inherent tissue characteristics, they may be at higher risk of occurrence. However, given the severity of bothersome symptoms and robust nature of the prolapse surgeries, it is often recommended to proceed with operative management after more conservative approaches have failed.

Patients with EDS often are affected by gastric dysmotility, highlighting the importance of having a gastroenterologist involved in patient care especially when considering surgery, where risk of postoperative gastrointestinal complications, including ileus, is higher. Defecatory problems are also greater in EDS groups, with significantly more patients reporting straining to move their bowels, feelings of incomplete defecation, and needing to perform digitation to defecate [15]. Constipation may be secondary to physical obstruction form the pelvic organ prolapse but is often multifactorial [15]. Constipation is common and usually requires sustained daily laxative use [8].

## 4. Conclusion

POP is unfortunately exceedingly common in the EDS population and often presents at a younger age and sometimes in nulliparous patients. POP in this cohort is by no means a straightforward issue that can be solved with supportive appliances or surgery alone. A multidisciplinary biopsychosocial approach that includes a team of dedicated physicians is required to provide safe and effective management of patients with bothersome POP and EDS. Expectations and goals should be discussed preoperatively between the patient and surgeon.

## Data Availability

Data will be provided on request.

## Abbreviations

POP: Pelvic organ prolapse
POP-Q: Pelvic Organ Prolapse Quantification
FPMRS: Female pelvic medicine and reconstructive surgery.
